# Recurrence of psoriasis on the resolution sites left with lentiginous pigmented patches after TNF inhibitor therapy^⋆^

**DOI:** 10.1016/j.abd.2022.04.015

**Published:** 2023-06-23

**Authors:** Toshiyuki Yamamoto

**Affiliations:** Department of Dermatology, Fukushima Medical University, Fukushima, Japan

Dear Editor,

To date, several cases of multiple lentigines in resolved psoriasis lesions have been reported. Herein, we describe a rare case of psoriasis that resolved leaving multiple small lentiginous patches in the lesions after successful treatment with a Tumor Necrosis Factor (TNF) inhibitor. Moreover, a recurrence of psoriasis was observed in the pigmentary patches.

A 55-year-old male was diagnosed with psoriasis vulgaris 6 years previously and had been treated with topical corticosteroid ointment. Joint pain appeared on the bilateral fingers, wrists, and ankles 3 years previously, and he received systemic therapy with adalimumab (subcutaneous injection of 80 mg, and 40 mg thereafter every other week). Both cutaneous and joint manifestations responded well to adalimumab. Psoriasis Activity and Severity Index (PASI) score was reduced from 6.0 to 0 (PASI clear), and also, he was relieved from joint pain. After the complete disappearance of psoriasis, pigmentation emerged. However, during maintenance therapy with adalimumab, cutaneous psoriasis relapsed 7 months later, but without recurrence of joint pain. Physical examination showed multiple brownish plaques on the lower extremities. A few psoriatic plaques were observed in some, but not all, of the resolved lesions (Fig. 1A‒C). In one of the lesions, psoriatic lesions appeared within the pigmented macule and spread beyond the pigmented macule (Fig. 1C).

**Figure 1:**
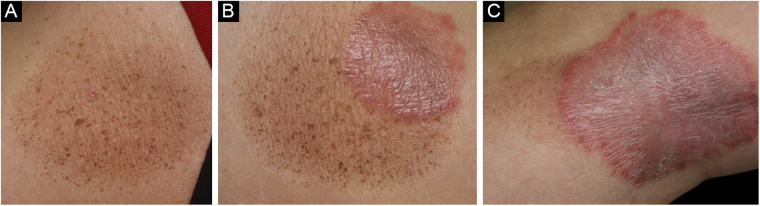
(A) A number of small brownish lentiginous patches appeared after improvement of psoriasis. (B) Psoriatic plaques were observed within part of the pigmentary macule. (C) Psoriasis occurred within the pigmentary macule and spread beyond the pigmentary macule

There are several cases of psoriatic plaques that were treated with biologics such as TNF inhibitors, Interleukin-17 (IL-17) inhibitors, IL-12/23 inhibitors, T-cell inhibitors, and phosphodiesterase 4 inhibitors, and left lentiginous lesions in the resolved area.^1^, ^2^, ^3^, ^4^ Previous studies have shown that inflammatory cytokines such as TNF-α and IL-17 can inhibit melanocyte growth, downregulate pigmentation-related gene expression, and render tyrosinase activity.^4^ Therefore, inhibitors of those cytokines may cause hyperpigmentation in susceptible individuals’ skin type and genetic predisposition. It is known that psoriasis arises in the resolved areas, which is considered to be an isomorphic response of Köbner. In the present case, scaly erythematous lesions recurred on some of the pre-existing lesions. The recurred psoriasis plaques did not perfectly but mostly correspond to the pre-existing areas. In another lesion, psoriasis initially recurred in the resolved areas and extended beyond the pigmentary macule. Previous studies showed that epidermal CD8+ T-cells increased in the Köbner-positive psoriasis skin, and CD8^+^ tissue Resident Memory T-cells (T_RM_) enriched in the resolved lesion preferentially produced IL-17 and IL-22 upon restimulation.^5^ However, triggering factors that stimulate T-cell activation are currently unknown. One possible cause may be the decrease in the efficacy of TNF inhibitors on cutaneous psoriasis. Unfortunately, we could not compare the frequency of epidermal T_RM_ on the non-lesional skin, improved skin with pigmentation, recurred psoriasis lesion in the resolved area, and recurred psoriasis lesion in the previously non-lesional area, because the patient refused biopsy. Further studies are necessary to elucidate the mechanism of lentiginous pigmentation after the improvement of psoriasis and the role of T_RM_ in such conditions.

## Financial support

None declared.

## Author' contributions

Toshiyuki Yamamoto: Study conception and planning; data collection, analysis and interpretation; management of studied cases; preparation and writing of the manuscript; approval of the final version of the manuscript.

## Conflicts of interest

None declared.
